# Targeting Herpes Simplex Virus Glycoprotein D with Bispecific Antibodies: Expanding Therapeutic Horizons by Searching for Synergy

**DOI:** 10.3390/v17020249

**Published:** 2025-02-12

**Authors:** Doina Atanasiu, Wan Ting Saw, Harvey M. Friedman, Gary H. Cohen

**Affiliations:** 1 Department of Basic and Translational Sciences, School of Dental Medicine, University of Pennsylvania, Philadelphia, PA 19104, USA; wsaw@upenn.edu; 2 Infectious Disease Division, Department of Medicine, Perelman School of Medicine, Penn Institute for RNA Innovation, University of Pennsylvania, Philadelphia, PA 19104, USA; hfriedma@pennmedicine.upenn.edu; 3 Department of Basic and Translational Sciences, School of Dental Medicine, Penn Institute for RNA Innovation, University of Pennsylvania, Philadelphia, PA 19104, USA; ghc@upenn.edu

**Keywords:** HSV, glycoproteins, neutralizing antibodies, recombinant antibodies, bispecific antibodies

## Abstract

Herpes simplex viruses (HSV-1 and HSV-2), which can be transmitted both orally and sexually, cause lifelong morbidity and in some cases, meningitis and encephalitis. While both the passive transfer of neutralizing antibodies and placental transfer of anti-HSV monoclonal antibodies (Mabs) have shown therapeutic promise in animal models, clinical trials have yet to identify approved immunotherapeutics for herpes infection. Here, we present strategies for the generation of recombinant bispecific antibodies (BsAbs) that target different domains of glycoprotein D (gD), crucial for HSV entry, that have the potential to outperform the effect of individual Mabs to curb herpes infection. Specifically, we selected three pairs of Mabs from our extensive panel for BsAb design and production based on their binding site and ability to block virus entry. Actual binding of BsAbs to gD and epitope availability on gD after BsAb binding were characterized using surface plasmon resonance (SPR) and inhibition by IgG Fab fragments generated from selected Mabs. While one BsAb exhibited an additive effect similar to that observed using a combination of the Mabs utilized for its generation, two showed antagonistic effects, suggesting that the simultaneous engagement of two epitopes or selective binding to one affected their activity against HSV. One BsAb (DL11/1D3) targeting the binding site for both nectin-1 and HVEM receptors demonstrated synergistic inhibitory activity against HSV, outperforming the effect of the individual antibodies. Recombinant DL11/1D3 antibody variants, in which the size of one or both paratopes was decreased to single chains (scFv-Fc), highlighted differences in potency depending on antibody size and format. We propose that BsAbs to individual glycoproteins offer a potential avenue for herpes therapeutics, but their design, mechanism of action, antibody format, and epitope engagement require careful consideration of structure for optimal efficacy.

## 1. Introduction

Herpes simplex viruses (HSV-1 and HSV-2) are double-stranded DNA viruses that can be transmitted both orally and sexually and cause lifelong mucocutaneous lesions, keratitis, and in some cases, meningitis and encephalitis (reviewed in [1,2]). Infection of neonates with HSV is also of particular concern, as it can lead to severe morbidity and mortality [3]. It is estimated that globally, 3.7 billion people under the age of 50 are infected with HSV-1 and 491 million people between the ages of 15–49 are infected with HSV-2 [4], the leading cause of genital herpes. In the USA alone, it is estimated that 40 to 60 million people are HSV-2 infected, with an incidence of 1–2 million infections and 600,000–800,000 clinical cases per year. Moreover, genital HSV-2 infections contribute to the spread of HIV (reviewed in [5,6]). The current drugs approved for the treatment of the herpes disease and the gold standard in a clinical environment include valacyclovir, cidofovir and foscarnet, which target viral DNA replication. However, long-term treatment with these drugs may lead to drug resistance in as many as 36% of people with a compromised immune system [7,8,9]. Therefore, there is a need to explore new strategies against drug-resistant HSV to identify new molecules that are highly effective and well tolerated.

Studies in animal models have demonstrated that the passive transfer of neutralizing antibodies confers prophylactic and therapeutic benefits [10,11,12] and the placental transfer of HSV-specific antibodies protects neonatal mice against HSV-2-associated mortality [13,14,15]. Current immunotherapeutic strategies to fight HSV infections in people have focused on the use of anti-HSV Mabs. However, while both UB-621 (E317), a Mab targeting glycoprotein D, and a gD-specific IgG1 isolated using a phage display library (HSV8) in combination with a broadly neutralizing anti-HIV antibody, were found to be safe and well tolerated in healthy individuals in phase I clinical trials [16,17], neither HSV Mab advanced to Phase II trials. However, a humanized anti-gB Mab, HDIT101(Mab 2c) is now in two Phase II clinical trials for both intravenous application and as a topical application in chronic recurrent orolabial HSV-1 infections [18,19]. Despite this recent advance, additional immunotherapeutics to treat the herpes disease are clearly needed [20,21,22,23,24]. Critically, the new NIH Strategic Plan [25] highlights the need for further research towards the development of novel antivirals such as Mabs “that can synergize with current regimens to enhance their function and potentially treat resistant infections and reduce shedding. Individual HSV Mabs can confer levels of protection and neutralization of HSV and may be a promising strategy to treat or prevent HSV infection”.

To address the critical need for immunotherapeutics and further understand the mechanism of action of Mabs and glycoproteins, we exploited our extensive knowledge and large panel of anti-HSV antibodies to generate unique bispecific antibodies (BsAb) against HSV [26,27,28,29,30]. The HSV envelope is decorated by at least ten glycoproteins and entry into host cells primarily relies on four: gD, gH, gL and gB (reviewed in [31,32,33,34,35]). The sequence of events leading to entry begins with the binding of gD to one of the cellular receptors (nectin-1 or HVEM), which causes conformational changes in gD. HSV gD then activates the heterodimer gH/gL to modulate fusion, which in turn, activates the fusogen, gB (reviewed in [32,36,37,38]). Notably, this process can be disrupted by specific antibodies that target any of the four fusion proteins driving the sequential stages of virus entry. Experimental and structural data show that neutralizing antibodies and those that block spread bind epitopes at or near the functional site of their target protein, thereby interfere with essential steps in the virus life cycle as well as in the cell–cell fusion pathway [16,39,40,41,42,43,44]. Understanding the mechanism by which a neutralizing antibody inhibits a specific step of virus entry, especially at the initial stages, is of high interest in vaccine development, immunotherapies and antiviral drug design.

Antibodies have traditionally been produced by hybridoma technology. However, these cells are sensitive and subject to poor recovery or loss after prolonged storage in liquid nitrogen. In contrast, the evolution of recombinant DNA technology that allows for the in vitro production of antibodies offers numerous advantages, including the ability to engineer antibodies with altered binding affinities and the generation of single chain (scFv-Fc) forms that can be delivered more efficiently. Finally, as demonstrated here, this strategy can be readily adapted to generate BsAbs that recognize multiple epitopes within the same antigen or epitopes on different antigens [45,46,47,48,49].

Here, we provide a proof of concept for the design and production of three recombinant gD BsAbs, each recognizing two distinct epitopes on gD. Crucial in their design was our evaluation of the effect that combinations of two antibodies have on cell–cell fusion and virus entry [50]. Our goals were the following: (1) to identify pairs of antibodies that have an additive or greater effect on cell–cell fusion; (2) to design BsAb by choosing Mabs that recognize non-competing epitopes on the surface of gD [28,29]; (3) to identify BsAbs with an activity that at least equaled the activity of the combination of single IgGs from which they were derived as potential tools for disease treatment; (4) to understand the differential effect of the combination of single IgGs vs. BsAb on gD function and conformational changes during virus entry and fusion. After screening multiple combinations of gD antibodies, we chose three pairs for BsAb design and production: two pairs (MC2+DL11; DL11+1D3) that had an additive effect on fusion and one combination (MC5+DL11) that had an antagonistic effect. Following the generation of BsAbs, we found that while MC5/DL11 was antagonistic as expected, MC2/DL11 BsAb was antagonistic rather than additive, and moreover, that the effects of DL11/1D3 BsAb were unexpectedly synergistic. Thus, the design of BsAbs requires knowledge of the properties of the original Mabs, such as the precise location of their epitopes, and a deep understanding of their mechanism of action. Other contributing factors in their design are the manipulation of the antibody structure (full IgG vs. single chain) for optimal efficiency, as well as preparing for the possibility of generating a “functional” competition when two recombinant antibodies are mixed.

## 2. Materials and Methods

### 2.1. Cells and Soluble Proteins

Mouse melanoma B78 were a gift from Meenhard Herlyn from the Wistar Institute (Philadelphia, PA, USA). Epithelial Vero African green monkey cells (ATCC, CCL-81) were grown in DMEMsupplemented with 5% FBS and 100 ug/mL penicillin-streptomycin. B78-C10 cells, stably expressing nectin-1 receptor, were grown in the same medium as B78 cells, with the addition of 500 µg/mL geneticin [51]. 293T cells were grown in DMEM supplemented with 10% FBS. HSV-1 KOS tk12 and HSV-2 (333) gJ beta-gal reporter viruses were generously provided by P.G. Spear [52,53]. Wild-type HSV-1 and HSV-2, as well as reporter viruses were grown and titered on Vero cells. gD_2_(285t) was purified from supernatants of mammalian transfected cells [54].

### 2.2. Plasmids

gB_1_ (PEP98), gD_1_ (PEP99), gH_1_ (PEP100), gL_1_ (PEP101) were gifts from Pat Spear [55] and Rluc8_(1–7)_ and Rluc8_(8–11)_ from Zene Matsuda [56,57]. gB_2_ (pTC580), gD_2_ (pTC578), gH_2_ (pTC510), gL_2_ (pTC579), gD P54Q (pDS90), gD S140N (pMM80) were all described previously [58,59,60,61,62,63,64].

### 2.3. Antibodies and Murine Fabs Preparation

All gD Mabs were previously published: 1D3 [65], DL11 [66], MC2, MC5, MC23 [29]. Fabs were isolated using a Pierce Fab Preparation Kit (Thermo Scientific, Rockford, IL, USA), according to the manufacturer’s protocol. Briefly, 5 mgs of murine IgG were incubated with 250 µL papain immobilized on agarose resin for 4 h at 37 °C. After digestion, Fab was purified using a Protein A spin column. Protein concentration was determined by absorbance at 280nm using an estimated extinction coefficient of 1.4.

### 2.4. Isotyping

All mouse IgGs were diluted to 100 ng/mL and isotyped using Pierce Rapid Isotyping Kit following the manufacturer’s protocol.

### 2.5. Hybridoma Sequencing and Generation of Antibody Expressing Plasmids

Hybridoma sequencing were performed by Genscript (Piscataway, NJ, USA). Total RNA was isolated from hybridoma cells and reverse-transcribed into cDNA using isotype-specific antisense primers or universal primers following PrimeScriptTM 1st Strand cDNA Synthesis Kit’s manual. Antibody fragments of V_H_, V_L_, C_H_ and C_L_ were amplified according to the standard operating procedure of rapid amplification of cDNA ends of GenScript. Amplified antibody fragments were cloned into a standard cloning vector separately. Colony PCR was performed to screen for clones with inserts of correct sizes. No less than five colonies with inserts of correct sizes were sequenced for each fragment. The sequences of different clones were aligned, and the consensus sequence was provided. Gene synthesis and direct cloning into pCDNA3.1 were performed with Genscript. All heavy chains were synthesized with a 6xHis or a Flag tag at the C-terminus or untagged, as indicated. For the generation of single chain antibodies, the variable regions from the heavy and light chains were linked with a (Gly_4_S)_3_ linker and anchored to mouse IgG2a Fc.

### 2.6. Expression and Purification of Antibodies

A total of 2 × 10^6^ 293T cells were seeded on each well of a 6-well plate and transfected the following day with 10 µL Lipofectamine 2000 and 1 µg each of the heavy (H_C_) and light chain (L_C_) plasmids in OptiMEM (Gibco, Grand Island, NY, USA). Then, 48 h later, supernatants were collected, and cells were refed with 1 mL of OptiMEM for another 48 h. Supernatants from both collections were pooled, clarified by centrifugation and filtered before dialysis with PBS overnight at 4 °C. The monospecific antibodies were purified on a ProG column (Genscript, Piscataway, NJ, USA). For bispecific antibodies, the dialyzed supernatants were incubated with “Ni-NTA superflow” nickel resin (Qiagen, Hilden, Germany), with gentle shaking for 24 h at 4 °C. After washing with PBS and wash buffer (10 mM imidazole, 20 mM phosphate (pH 7.5), 500 mM NaCl) the Abs were eluted with elution buffer (20 mM, 50 mM and 250 mM imidazole, 20 mM phosphate (pH 7.5), 500 mM NaCl).

### 2.7. Western Blotting

For evaluation of antibody production, 100 ng of purified antibody were run on a Novex 10% Tris-Glycine gel under “native” conditions [66]. Blots were probed with goat anti-mouse/or anti-human peroxidase and developed using Pierce substrate.

For glycoprotein recognition, 100 ng of gD_2_306t were run on 10% Tris-Glycine gels under native conditions. After transfer, nitrocellulose membranes were probed with 1 µg/mL purified antibodies as indicated.

### 2.8. Surface Plasmon Resonance (SPR)

Experiments were performed using a Biacore 3000 biosensor (Cytiva, Marlborough, MA, USA), at room temperature. Filtered and degassed HBS-EP buffer (10 mM HEPES (pH 7.4), 150 mM NaCl, 3 mM EDTA, 0.005% surfactant P20) was used in all the experiments. Anti-His (Qiagen, Inc., Germantown, MD, USA) was covalently coupled to a CM5 sensor chip (Cytiva, Marlborough, MA, USA) following our previous protocol [33]. Then, 150–200 resonance units (RUs) of wt gD_2_285t were captured. Purified mouse and recombinant IgGs were then injected for 300 s. After each experiment, the chip surface was treated with brief pulses of 0.2 M Na_2_CO_3_ (pH 11) until the RU signal returned to baseline, and then a new cycle was started. For epitope availability experiments, 5 µg/mL of bispecific antibodies were captured to a ProA chip (Cytiva, Marlborough, MA, USA). Then, 150–200 RUs of wt gD_2_285t were flowed. A total of 50 ug/mL of purified Fabs were then sequentially flowed. The surface of the chip was regenerated with 10 mM glycine-HCl pH 1.5. All injections were performed at a flow rate of 5 μL/min.

### 2.9. Split Luciferase Assay

B78 and C10 cells were plated in preparation for transfection based on previously described protocols [58,67]. B78 cells plated on a 96-well plate were transfected with 40 ng each of gB, gD, gH, gLand Rluc8_(1–7)_. C10 cells were plated on 6-well plates. Each well was transfected with 1 µg each of HVEM and the Rluc8_(8–11)_ plasmid. Twenty-four hours post-transfection, B78 cells were pre-incubated with 5 µg/mL of the indicated Mabs or 10 µg/mL of the BsAb for one hour. Fusion was triggered by the addition of nectin-1/HVEM cells to the B78 cells. Reconstitution of luciferase was monitored for 2 h, with readings taken every 5 min, using a BioTek plate reader. Blocking activity of antibodies was expressed as the percentage of cell–cell fusion compared to fusion in the absence of Abs (100%) to avoid variability and increase in background noise.

### 2.10. β-Galactosidase Reporter Assay for HSV Entry

Confluent Vero cell monolayers were grown in 96-well plates and infected at multiplicity of infection (m.o.i) of 1 with HSV-1 or HSV-2 β-gal reporter viruses. Viruses were pre-incubated with the Mabs at the indicated concentrations for 1 h at 37 °C before addition to the Vero cells. After 6 h, cells were lysed with 0.1% Nonidet P-40 (Sigma, St. Luis, MO, USA) and CPRG (chlorophenol red-β-d-galactopyranoside, Roche Diagnostic, Indianapolis, IN, USA) was added. The β-galactosidase activity was read at 595 nm with a BioTek plate reader. β-galactosidase activity indicated successful entry. Blocking activity of antibodies was expressed as the percentage of virus entry into cells compared to entry in the absence of antibodies (100%).

### 2.11. Plaque Assay

One hundred plaque forming units of wt HSV-1 or HSV-2 were pre-incubated with Mabs at the indicated concentrations for 1 h at 37 °C. The mixture was added to Vero cells for an additional hour at 37 °C Monolayers were then overlaid with 1% methylcellulose and incubated with the Mab-virus mixture for 2–3 days, until plaques were visible. Cells were fixed with 5% formaldehyde solution. Plaques were stained with crystal violet and counted.

### 2.12. Evaluation of Effect of Combination Antibodies

The Bliss independence model [68] was applied. The activity of each individual Mab was used to calculate a theoretical additive curve using the formula for probabilistic independence:

E_A_ + E_B_(1 − E_A_) = E_A_ + E_B_ − E_A_E_B_.

### 2.13. Statistical Analysis

Two-tailed Student *t* test (GraphPad Prism 9.5.1) was used to determine *p* values.

## 3. Results

### 3.1. Screening Candidates for Bispecific Antibodies

We previously evaluated the effects of a limited number of gD Mab combinations on cell–cell fusion, with a focus on the interaction between gD and gH/gL [50]. The contribution of two Mabs to the inhibition of fusion was evaluated using the Bliss independence model [68]. This model assumes that two inhibitors (antibodies) with independent binding sites and independent mechanisms of action will act in such a manner that neither of them interferes with each other but both contribute to the overall result [68].

**Figure 1:**
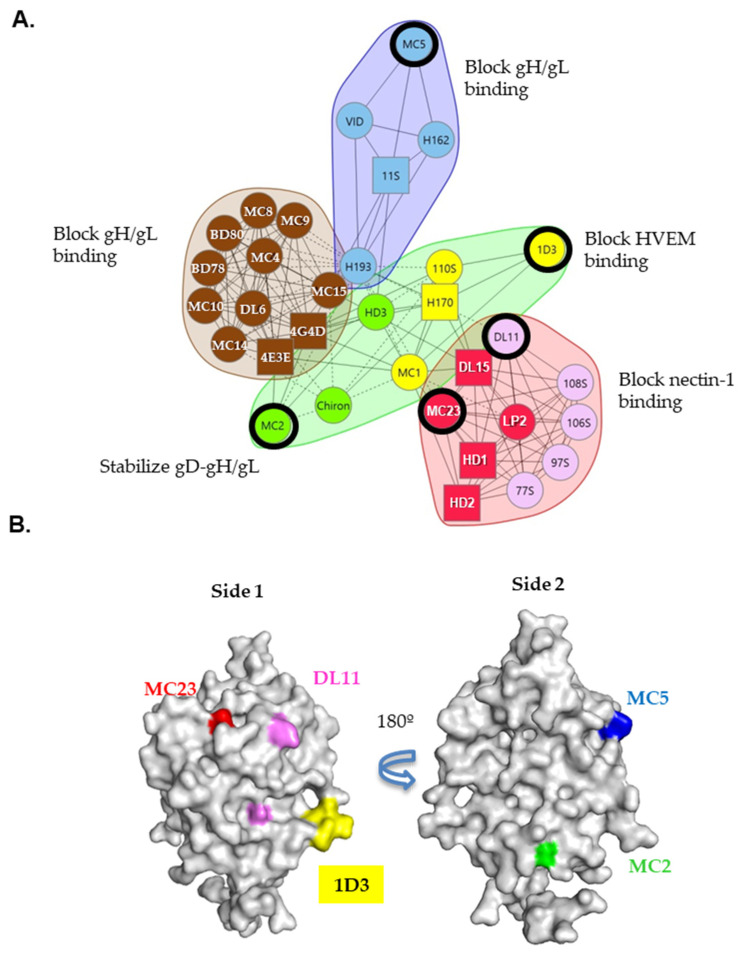
Epitope location and community map of gD monoclonal antibodies. (**A**) Organization of gD antibodies in communities based on their competition for binding to gD. Antibody names in a sphere indicate that competition was measured as both ligand and analyte; squares indicate that competition was measured as either a ligand or an analyte. Bidirectional competition is indicated by solid connecting lines. Dashed connecting lines identify unidirectional competition. Black rings highlight key neutralizing Mabs used in this study. (**B**) Epitope location of sentinel gD monoclonal antibodies (colored) is indicated on the crystal structure of gD285 (PDB 2C36). Side 1 contains epitopes important in the gD-receptor interaction: MC23 (nectin-1), 1D3 (HVEM) and DL11 (nectin-1). Side 2 is involved in post-receptor binding events, including the interaction with gH/gL. MC5 (blue) blocks while MC2 (green) stabilizes the gD-gH/gL interaction.

Here, we have expanded our analysis to include other neutralizing gD Mabs. Using competition analysis, we organized these Mabs into a community map ([28] and 1A). Mabs from the same community inhibit virus entry through similar mechanisms: blocking the binding of the virus to nectin-1 receptor (Mabs from red and pink community), blocking binding to HVEM (yellow), blocking of the gD-gH/gL interaction (red, blue and brown) or stabilization of the gD-gH/gL interaction (green). In this study, we aimed to identify pairs of Mabs that have an additive inhibitory effect to advance our design of therapeutic BsAbs, using the Bliss model and a cell–cell fusion assay as a screening tools. Specifically, we selected non-competing, neutralizing Mabs MC2, MC5, MC23, DL11 and 1D3 as representatives of the green, blue, red, pink and yellow communities, respectively (Figure 1 and Table 1). Apart from MC2 (which only binds gD from HSV-2), all Mabs are type common.

To determine the effect of two Mabs, we modified our split luciferase assay [58,67] in which successful fusion results in the reconstitution of luciferase, allowing us to monitor the inhibitory effects of the Mabs on the overall fusion process. Effector B78 cells expressing gB, gD, gH/gL and half of the split renilla luciferase were pre-incubated with 5 ug/mL of each Mab (singly or in combination) for 1 h at 37 °C. Target C10 cells contain the other half of the split renilla luciferase, express nectin-1 and were engineered to also express HVEM to monitor the ability of Mabs to block gD binding to either nectin-1 (DL11, MC23) or HEVM (1D3 Mab) (Table 1; [62,69]). Fusion was triggered by the addition of nectin-1/HVEM target cells to the effector cells. The reconstitution and production of luciferase was monitored as a readout for fusion for 2 h with a BioTek plate reader. The blocking activity of the different antibodies was normalized to “no antibody” samples.

**Figure 2:**
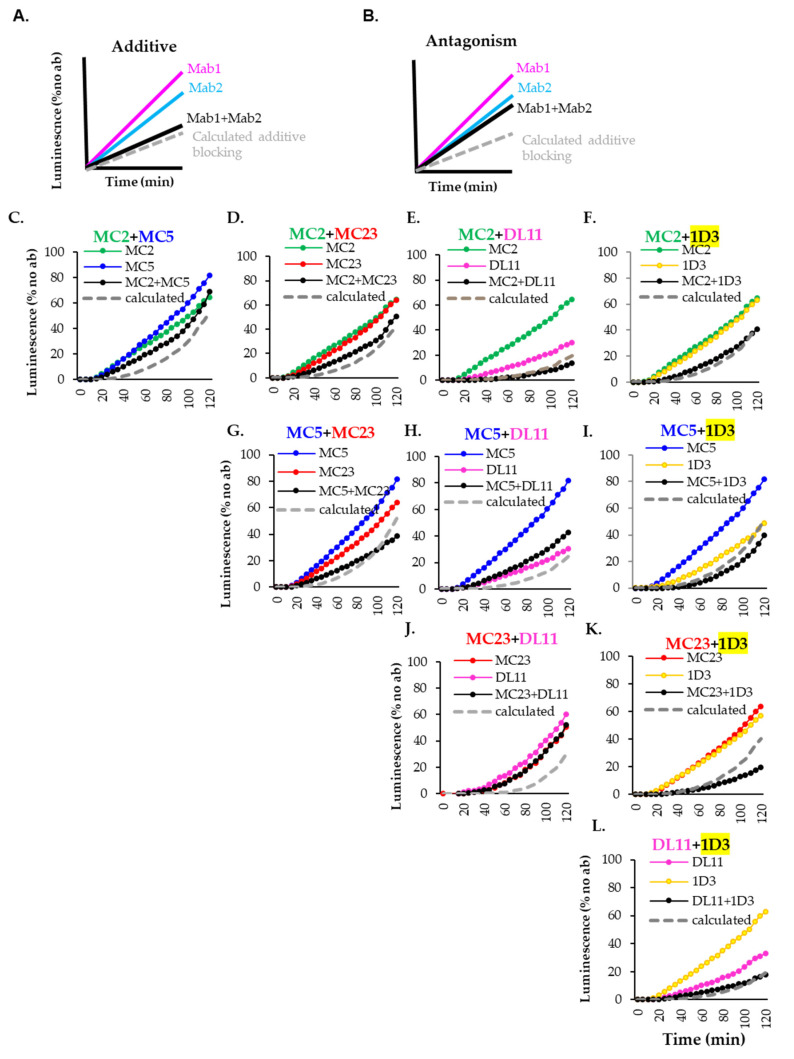
Screening of pairs of murine monoclonal antibodies for use as candidates for bispecific antibody design. When combinations of non-competing antibodies are used in cell–cell fusion, a theoretical, additive blocking curve (gray) can be calculated based on the ability of each Mab (colored curves) to block fusion. There are two possible outcomes: additive effect (**A**), when the combination of Abs is as good as a theoretical additive curve, or antagonistic (**B**), when the combination is only as good as the more active Ab. (**C**–**L**) Non-competing gD antibodies were evaluated for their blocking activity in a split luciferase assay. The effect of two antibodies (black curves), each at 5 µg/mL was evaluated over a 2 h time course. Each experiment was performed at least three times, each in duplicate. Representative experiments are shown.

To analyze the combined effects of the two Mabs, we used the Bliss independence model [50,68] to calculate a theoretical additive curve (Figure 2, gray dashed curves) based on the inhibitory effect of each Mab. This calculation assumes that both Mabs block in an additive way. A close overlap between our measured (black) and the theoretical curve (gray) indicates that a combination of the two Mabs has an “additive effect” due to independent mechanisms of action in fusion (Figure 2A). Divergence of the measured and theoretical curves, with the measured curve more closely resembling that of the more active Mab, indicates that the combination is “indifferent”/antagonistic (Figure 2B) and that the two Mabs competed or inhibited the same functional step in fusion. We expected that non-competing Mabs that blocked different steps of the interaction of glycoproteins leading to fusion (see Table 1) would have an additive effect while those that competed for binding to gD (such as MC23+DL11, [28,29,70]) or have a similar inhibition mechanism (MC2+MC5, [71]) would have an “indifferent” effect.

Figure 2 shows the inhibitory effect of the five Mabs (colored curves) and all the possible combinations (black curves). Results are summarized in Table 2. As anticipated, the combination of competing Mabs MC23+DL11 (Figure 2J) or Mabs that block the same step (MC2+MC5, Figure 2C) in the fusion cascade [71] had an “indifferent” effect and were only as good as the single Mabs. In contrast, combinations of non-competing Mabs that inhibit fusion through different mechanisms had an additive effect: MC2+DL11 (Figure 2E), MC2+1D3 (2F), MC5+1D3 (2I), MC23+1D3 (2K), DL11+1D3 (2J).

Despite our predictions, three combinations had an unexpected effect: MC2+MC23 (Figure 2D), MC5+MC23 (Figure 2G) and MC5+DL11 (Figure 2H). First, MC2 and MC23, which were presumed to have non-overlapping inhibitory mechanisms (MC2 stabilizes gD-gH/gL interaction and MC23 blocks gD binding to gH/gL and nectin-1), and would, therefore, be additive, were surprisingly found to be “indifferent” (Figure 2D). Second, although both MC5 and MC23 Mabs block the gD-gH/gL interaction [70], and were, therefore, expected to have an “indifferent” effect, we found them to be additive. Third, MC5 and DL11 do not compete for the same epitope and were shown to block different steps in the fusion pathway: the gD-gH/gL interaction (MC5) and nectin-1 binding (DL11). Surprisingly, this combination was also found to be “indifferent” (Figure 2H). This suggests that despite the wealth of knowledge about their role in viral infection [28,39,50,69,70,71,72,73,74,75], certain aspects regarding the mechanism of action of MC2, MC5 and MC23 Mabs are still elusive. However, from our analysis, we can conclude that combinations of non-competing gD Mabs that differ in their mechanism of blocking will have an additive blocking effect on fusion, as previously published [50].

### 3.2. Isotyping, Sequencing and Cloning

**Figure 3:**
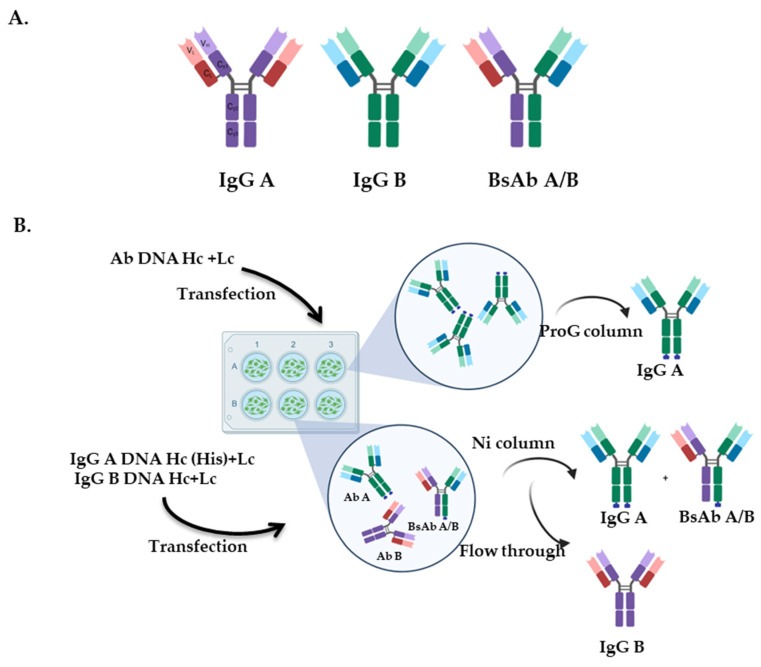
Schematic representations of recombinant antibodies. (**A**) Immunoglobulin G (IgG) is composed of a fragment crystallizable (Fc) region and two fragment antigen binding (Fab) arms. A bispecific antibody has two distinct paratopes and thus, is capable of binding two different epitopes within the same antigen or two different antigens. (**B**) Diagram of the expression and purification of recombinant antibodies. 293T cells were transfected with plasmids encoding for heavy (Hc) and light chains (Lc) to generate monospecific IgG A or IgG B. To generate a BsAb, A/B Hc+Lc for IgG A and Hc+Lc for IgG B were co-transfected. Monospecific antibodies were purified using ProA columns. BsAbs were purified on Nickel columns.

To streamline the efficiency of producing immunotherapeutics utilizing pairs of Mabs, we set out to determine if we could generate recombinant BsAb (Figure 3A) that had similar additive inhibitory activity as the combination of the two Mabs from which they were generated. Based on our analyses, we selected two additive pairs as candidates: MC2+DL11 and DL11+1D3. We also included the MC5+DL11 pair as an example of Mabs that, according to our prediction, would generate an antagonistic BsAb due to their functional competition.

To generate the recombinant Abs, we isotyped (Table 1) and sequenced the variable regions of the immunoglobulin heavy (Hc) and light chain (Lc) of the four hybridomas currently used to produce our Mabs (Supplemental S1). The corresponding DNA sequences were cloned into the pcDNA3 expression vector, as described in Materials and Methods. All Hc variable regions were cloned into a human IgG1 and all Lc variable regions into human kappa chains. For BsAb purification purposes, we added tags to the C-term of some of the heavy chains: 6xHis (MC2, MC5) or Flag (1D3).

### 3.3. Expression and Characterization of Recombinant Antibodies

**Figure 4:**
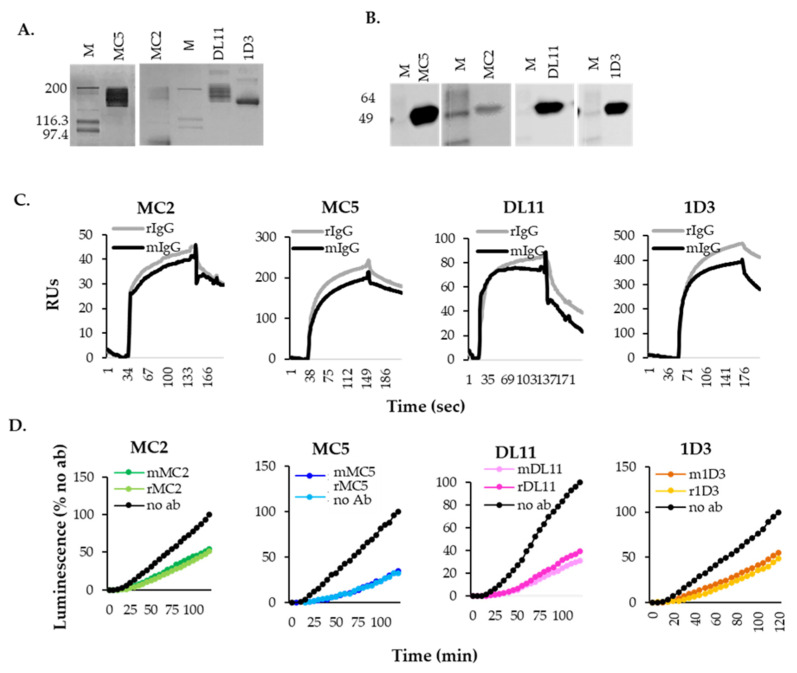
Purification and characterization of recombinant antibodies. (**A**) Silver staining. MC2, MC5, DL11 and 1D3 Abs were purified from 293T transfected cells using ProG columns. Proteins were run under native conditions. (**B**) Western blotting. All Abs recognized purified soluble gD_2_(285) run under native conditions. (**C**) gD_2_285t was immobilized on CM5 chip surface. Mouse (black) and humanized recombinant (gray) antibodies were flowed over the chip. (**D**) Comparison of blocking activity of recombinant humanized IgGs (lighter curves) and murine antibodies (darker curves) in a cell–cell fusion. Each antibody was used at a concentration of 5 µg/mL and their effect was evaluated over a 2 h time course. Three independent experiments performed in duplicate. Representative experiments are shown.

To generate recombinant versions of the original Mabs, 293T cells were transfected with plasmids encoding the Hc and Lc of MC2, MC5, DL11 or 1D3 plasmids (Figure 3B). Supernatants were collected and the recombinant Mabs (rMabs) were purified using ProG columns. Silver staining of purified rMabs showed that they run at the expected 150 kDa size (with the exception of MC2, which also showed a 75 kDa band) and were pure (Figure 4A). Furthermore, all rMabs recognized purified gD protein by Western blotting under native conditions (Figure 4B).

To further characterize the rMabs, we used surface plasmon resonance (SPR) to compare their binding properties to those of the original mouse IgGs. First, soluble gD_2_285t was amine-coupled directly to a CM5 chip. Because MC2, MC5, DL11 and 1D3 do not compete for binding to gD and bind non-overlapping epitopes [28,29], they can be sequentially flowed over the surface of the chip. Over one flow cell, we sequentially flowed mouse MC2, MC5, DL11 and 1D3 IgG (mIgG) over captured gD_2_. Using a second flow cell, we flowed the recombinant Abs (rIgG). In both flow cells, we observed an increase in mass, indicative of antibody binding. Figure 4C shows the comparison of binding of recombinant and murine antibodies to gD_2_285t. All rMabs displayed similar on/off rates to the corresponding mMab. These experiments showed that the recombinant humanized and murine IgGs were equivalent in their recognition and binding capacity to gD_2_285t.

We then tested the biological activity of the rMabs in a cell–cell fusion assay (SLA). B78 cells transfected with gB, gD, gH, gL and half of the reporter Rluc8_(1–7)_ were pre-incubated with 5 µg/mL of murine or their respective rMab. Fusion was triggered by adding nectin-HVEM expressing cells transfected with the other half of the reporter Rluc8_(8–11)_. As expected, the activity of recombinant MC2, MC5, DL11, 1D3 (Figure 4D, lighter colored curves) was indistinguishable from that of the original murine Mabs (darker colors) in fusion inhibition. We conclude that rMabs exhibit the same physical and biological properties in cell–cell fusion as the mMab from which they were derived, despite differences in their Fc regions.

### 3.4. Expression and Purification of BsAbs

To obtain BsAbs, 293T cells were co-transfected with the heavy and light chains of MC2_His_ and DL11 (for the generation of MC2_His_/DL11 BsAb), MC5_His_ and DL11 (MC5_His_/DL11 BsAb), and DL11_His_ and 1D3_Flag_ (DL11_His_/1D3_Flag_ BsAb). The light chains could randomly pair with any heavy chain, which theoretically could lead to the formation of multiple versions of the BsAb with different activities and reactivities. However, we have found that >90% of the activity of MC2 and MC5 and >60% for DL11 and 1D3 Abs relies on the heavy chain variable regions. This suggests that the light chain mispairing may not affect the activity of the BsAb population. For simplicity, we chose to portray the BsAb as an antibody with the correct pairing of the heavy and light chains (Figure 3A).

The supernatants from the transfected cells would contain three antibody populations. For example, after transfection with MC2_His_ and DL11 heavy and light chains, the supernatant would contain MC2_His_ IgG, DL11 IgG and BsAbs (Figure 3B). A purification of the supernatants using a ProG column will select all the Fcs and not distinguish between the two singles (MC2 and DL11) and the BsAb. However, a one-step purification through a nickel column would at least eliminate the untagged, free DL11 IgG and retain the antibody species containing MC2_His_ (free MC2 IgG and MC2/DL11 BsAbs). We hypothesized that if we could identify DL11 in this mix, then it must be part of MC2/DL11 BsAb. A similar approach could be applied for MC5_His_/DL11. For DL11_His_/1D3, we would need to show the presence of 1D3 to demonstrate the existence of BsAbs in the antibody mix.

**Figure 5:**
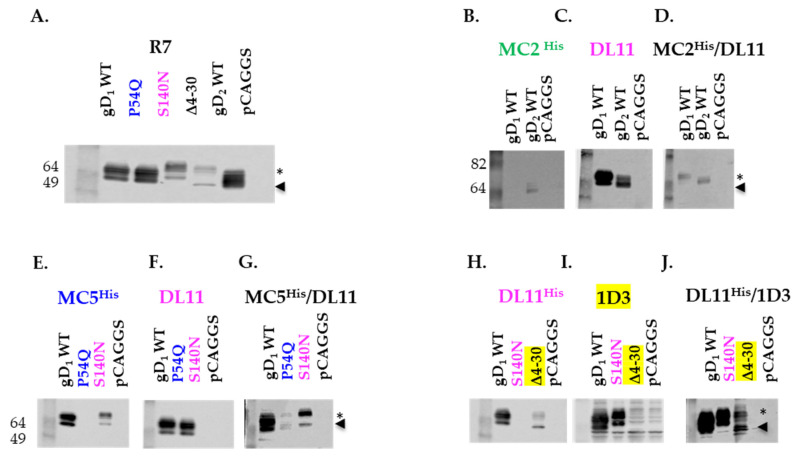
Characterization of recombinant antibodies. B78 cells were transfected with wt or mutant gDs that are resistant to specific antibodies: P54Q does not react with MC5 (blue), S140N is resistant to DL11 (pink) and Δ4–30 is resistant to 1D3 (yellow). Total cell lysates were separated by SDS electrophoresis under native conditions and probed with the indicated recombinant Abs. (**A**) A gD polyclonal antibody (R7) recognizes both mature (star) and precursor (arrowhead) gD. (**B**) Type-2-specific MC2 recognizes gD_2_ only, while type common DL11 reacts with both gD_1_ and gD_2_ (**C**). MC2/DL11 BsAb recognizes both gD_1_ and gD_2_ (**D**). MC5 (**E**) and DL11 (**F**) do not recognize mutants P54Q or S140N, respectively, but the MC5/DL11 reacts with both (**G**). Similarly, DL11 (**H**) and 1D3 (**I**) do not react with mutants S140N or Δ4–30, respectively, but the bispecific DL11/1D3 (**J**) reacts with both.

To show the presence of DL11 in MC2/DL11 and MC5/DL11, as well as the presence of 1D3 in DL11/1D3, we took advantage of the known properties of each homologous antibody to recognize specific gD mutants. We used mutant P54Q, which is unable to react with MC5 [71], S140N, which does not react with DL11 [61], Δ4–30, which removes the 1D3 epitope, wt gD_1_ (does not react with MC2) and wt gD_2_. B78 cells were transfected with the indicated constructs or empty vector (pCAGGS) as a negative control. Total cell lysates were prepared and run on 10% Tris gels under native conditions. After transfer, the nitrocellulose membranes were probed with R7 polyclonal Ab to detect gD production. All constructs produced full-length gD and the polyclonal Ab recognized both the mature and precursor protein (marked with a star and arrowhead, respectively, in Figure 5A). gD_1_, carrying the S140N mutation, runs at a higher molecular weight than wt gD_1_ due to the introduction of a glycosylation site [61], and the expression of the Δ4–30 mutant was lower than the other forms of gD.

**Figure 6:**
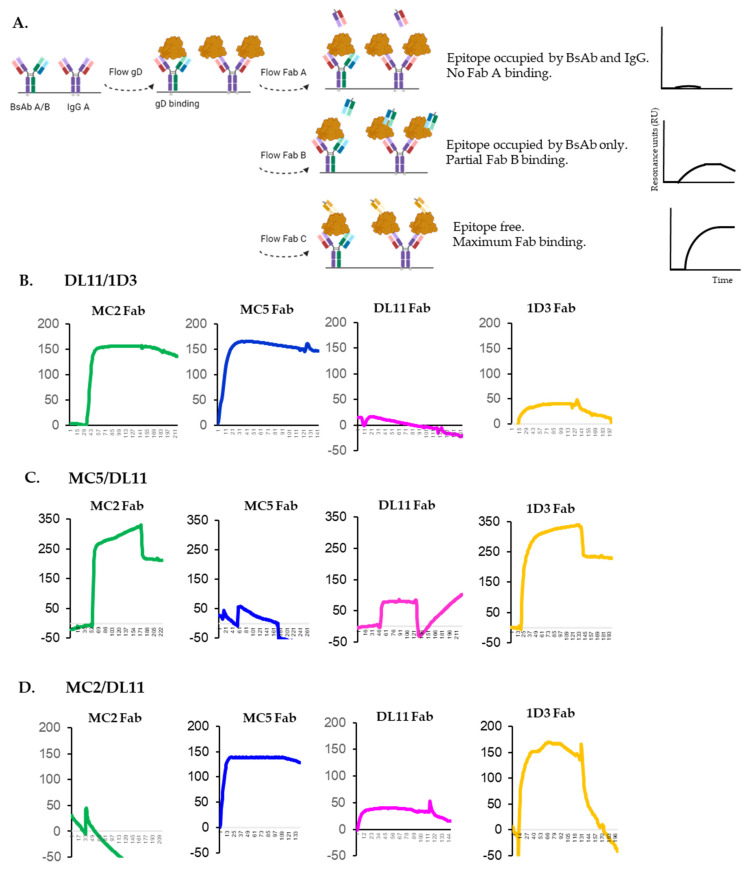
Epitope availability on gD after binding BsAbs. (**A**) Diagram of experimental set up. Bispecific antibodies DL11/1D3 (**B**), MC5/DL11 (**C**) or MC2/DL11 (**D**) were immobilized directly on a ProA chip. Next, soluble, purified gD_2_(285t) was flowed followed by sequential flowing of non-competing Fabs MC2 (green), MC5 (blue), DL11 (pink) and 1D3 (yellow). Only Fab binding is shown. Three independent experiments were performed. Representative curves are shown.

- MC2/DL11. Lysates from cells transfected with full length gD_1_ and gD_2_ were run under native conditions. After transfer, the nitrocellulose membranes were probed with purified rMC2 (Figure 5B), rDL11 (Figure 5C) or MC2/DL11 BsAb (Figure 5D). Due to its type-2 specificity, we expected that MC2 would only recognize gD_2_ [29] and DL11, a type common antibody, would bind both gD_1_ and gD_2_ [28,61]. MC2/DL11 should recognize both gD_1_ and gD_2_, due to the presence of DL11. We found that rMC2 recognized gD_2_, but not gD_1_ (Figure 5B) and DL11 reacted with both gD_1_ and gD_2_ (Figure 5C). MC2/DL11 BsAb recognized gD_1_ and gD_2_ suggesting that there is DL11 and only as part of the MC2/DL11 BsAb. The proportion of free IgGs in the purified BsAbs population will be discussed later (Figure 6).
- MC5/DL11. To determine the presence of DL11 as part of this BsAb, we used P54Q (MC5 resistant) and S140N (DL11 resistant) gD mutants [61,71]. Figure 5E shows that rMC5 did not react with P54Q and rDL11 did not recognize S140N (Figure 5F) as expected [61,71]. However, all gD constructs reacted with the BsAb sample, suggesting that there is DL11 as part of the MC5/DL11 Ab.
- DL11/1D3. For this Ab, we used the S140N gD (DL11 resistant) and a new mutant, Δ4–30, which removes the 1D3 linear epitope. As expected, the Δ4–30 and S140N mutants did not react with 1D3 (Figure 5H) or DL11 (Figure 5I). However, they did react with DL11/1D3 BsAb (Figure 5J). This suggested that 1D3, as part of the DL11/1D3 BsAb, is present.

We conclude that gD mutants resistant to MC5, DL11 and 1D3 Abs can be successfully used to demonstrate the presence of BsAbs after a one-step purification of supernatants from 293T transfected cells.

### 3.5. Epitope Availability of gD After Binding of Bispecific Antibodies

Next, we sought to determine the ratio of BsAb/free his-tagged IgG in these preps as well as understand the mechanism of action of the three recombinant BsAbs and how they bind gD. For this, we used SPR and Fabs of each of the respective antibodies to probe the surface of gD post BsAb binding. We assumed that if a hypothetical BsAb A/B is pure and engages both paratopes, then neither Fab A, nor Fab B would be able to bind gD, as the sites would already have been occupied. Accordingly, a third, non-competing Fab, Fab C, that was not part of the BsAb footprint, would bind, as the site would be available (Figure 6A). If the BsAb A/B prep also contains some free IgG A, then the epitope for Fab A will be completely occupied on all gD molecules, regardless of whether gD is bound by IgG or BsAb. This will result in no Fab A binding. The epitope for Fab B would be occupied on gDs bound to BsAbs but still be open on gDs bound by the free IgG A. This will result in some Fab B binding. The resonance units (RUs) measured for Fab B binding would reflect the levels of free IgG A. The epitope for Fab C will be open on all gDs, regardless of whether gD is bound by free IgG or BsAb. The binding of Fab C would represent the maximum RUs of Fab binding to gD in a particular experiment. This value, combined with the RUs measured after the binding of Fab B, can be used to estimate the % of free IgG A in the BsAb prep.

For this evaluation, we directly bound the BsAbs-IgG mix to a ProA sensor chip (Cytiva) and then flowed gD_2_285t over it (Figure 6A). Because they do not compete, Fabs MC2, MC5, DL11 and 1D3 were then sequentially flowed and their binding was evaluated.

(a) Estimation of free DL11 IgG in DL11/1D3 prep (Figure 6B). The MC2 (green curve) and MC5 (blue) epitopes were still available after gD bound the BsAb. The DL11 Fab (pink) did not bind and the binding of 1D3 Fab (yellow) was severely decreased compared to the binding of the non-competing MC2 and MC5 Fabs. The simultaneous occupancy of DL11 and 1D3 proves the presence of DL11/1D3 BsAb. We consider the residual binding of 1D3 Fab as a measure of the amount of free DL11 in the BsAb prep (Figure 6A). Using the binding of MC2 or MC5 Fabs as a measure of the total Fab binding to gD in this experiment (~150RUs), and based on the 1D3 Fab binding (~30 RUs), we estimate that the DL11/1D3 prep contains ~20% free DL11 IgG.

(b) Estimation of free MC5 IgG in MC5/DL11 prep (Figure 6C). After gD was bound by the Abs, we found that the epitopes for MC2 (green curve), 1D3 (yellow) and partially DL11 were available as indicated by the ability of the respective Fabs to bind gD. MC5 Fab (blue) did not bind. This suggested the following: (1) as expected, non-competing Fabs (MC2 and 1D3) could still bind gD at the sites not occupied by the Abs and (2) only the MC5 site was fully occupied (MC5 Fab did not bind gD), while the DL11 epitope was still partially open (Fab still bound gD). Based on the 1D3 (~320 RUs) and DL11 (80 RUs) Fabs binding, we estimate that there is ~25% free MC5 IgG in purified MC5/DL11.

(c) Estimation of free MC2 IgG in MC2/DL11 (Figure 6D). MC2 Fab did not bind gD (green curve), suggesting that this epitope was already occupied by the Abs. However, MC5 (blue), 1D3 (yellow) and partially DL11 (pink) Fabs all bound to gD. This suggested that not all DL11 sites were occupied by the BsAb. Using the binding of MC5 or 1D3 Fabs as a measure of the total Fab binding to gD in this experiment (~150 RUs), and the DL11 Fab binding (~40 RUs), we estimate that the MC2/DL11 prep contains ~25% free MC2 IgG.

We conclude that although the BsAbs were purified through one column only, they are at least 70% pure. We propose that the remaining 20–25% of free IgGs would not significantly contribute to the activity of the BsAbs or impact the interpretation of results.

### 3.6. Blocking Activity of Recombinant Antibodies in Cell–Cell Fusion

**Figure 7:**
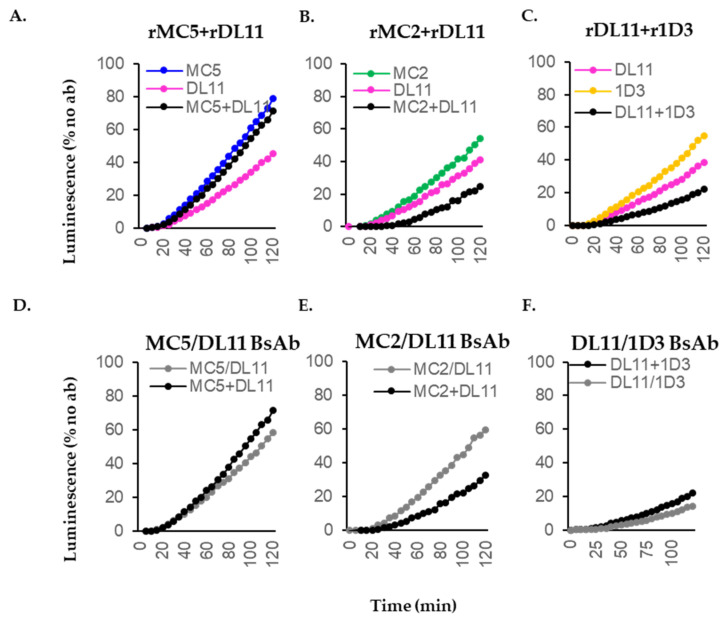
Blocking of cell–cell fusion by recombinant antibodies. (**A**–**C**) Effect of combinations of recombinant Abs (black curves) on cell–cell fusion over a 2 h time course compared to monospecific Abs at the same concentrations (colored curves). (**D**–**F**) Comparison of combinations of recombinant IgGs (black curves) and bispecific antibodies (gray curves) in cell–cell fusion. Each antibody was used at a concentration of 5 µg/mL, either single or in combination. BsAbs were used at 10 µg/mL. At least three independent experiments were performed, each in duplicate. Representative graphs are shown.

Next, we compared the activity of the combinations of rMabs with the activity of their respective BsAbs. We expected that the rMabs will have similar activities as the mMabs (Figure 2). Furthermore, the additive combinations rMC2+rDL11 (Figure 7B) and rDL11+r1D3 (Figure 7C) would translate into BsAbs with an activity at least equal to the combinations, providing that the distance between the two epitopes does not exceed the 6–12 nm coverage of the paratopes [76]. In contrast, the “indifferent” rMC5+rDL11 pair (Figure 7A) would form an antagonistic BsAb. Indeed, the MC5/DL11 inhibited fusion to the same extent as the rMC5+rDL11 combination (compare black and gray curves in Figure 7D) and was antagonistic. However, the MC2/DL11 BsAb was less active than the combination of Mabs (Figure 7E) and DL11/1D3 BsAb was as good as the additive rDL11+r1D3 combination (Figure 7F). Replicate experiments are shown in Supplemental Figure S1.

We conclude that rMabs exhibit the same biological properties in cell–cell fusion as the Mabs from which they were derived. Furthermore, combinations of antibodies that have an “indifferent” blocking effect will only generate an antagonistic BsAb. rMabs that have an additive effect will form an additive BsAb, as shown for DL11 and 1D3 Abs, which were the best performing BsAb. However, an additive combination can generate an antagonistic BsAb, suggesting that the distance between the two epitopes is not optimal [76,77], as may be the case for MC2/DL11. Consistency in the activity of the BsAbs between batches suggests that at this stage in our exploratory study, the proportions of antibodies in the BsAb mix are maintained.

### 3.7. Blocking Activity of Recombinant Abs Against HSV-2 Virus

To test the activity of all recombinant antibodies against the virus, we used two methods: virus penetration and plaque assays. In both assays, we used only HSV-2 to accommodate for the HSV-2 specificity shown by MC2 [29].

(a) Virus penetration (entry) assay. HSV-2 (333) βGal reporter virus was pre-incubated with two-fold dilutions of rMabs (singly or in combination) for 1 h. The virus–antibody mix was then added to Vero cells monolayers for 6 h. After lysis, CPRG substrate was added, and the β-galactosidase activity was measured as a surrogate for virus activity.

**Figure 8:**
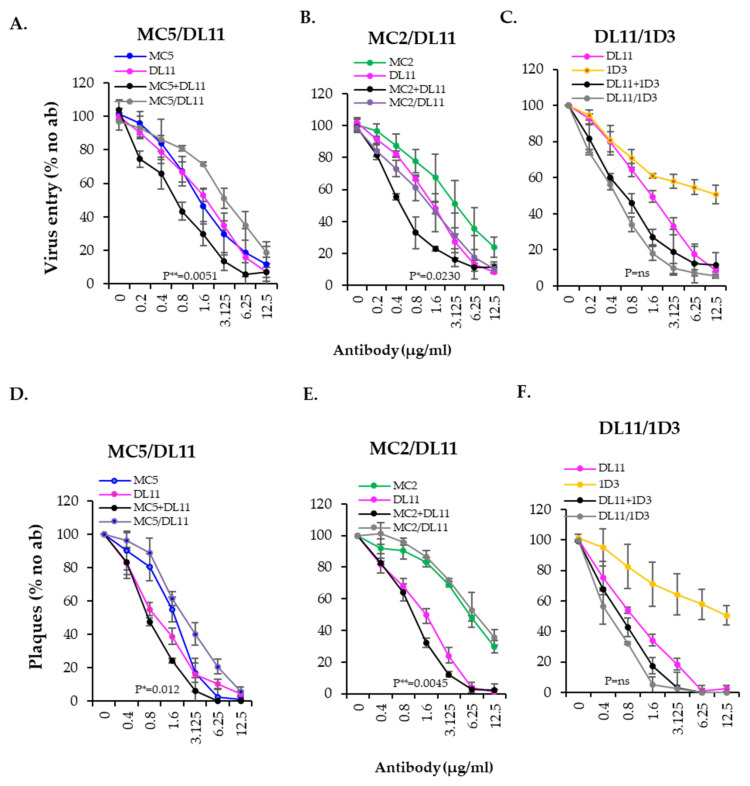
Inhibition of virus activity by recombinant antibodies. Effect of MC5/DL11 (**A**), MC2/DL11 (**B**) or DL11/1D3 (**C**) BsAbs on HSV-2 (333) β-galactosidase reporter virus infection of Vero cells using an entry assay. Virus was incubated with 2-fold dilutions of single IgGs starting at 6.25 µg/mL (single or in combination) or 12.5 µg/mL (BsAbs). Effect of MC5/DL11 (**D**), MC2/DL11 (**E**) or DL11/1D3 (**F**) BsAbs on HSV-2 infection in a plaque assay. Antibodies were used at 6.25 µg/mL (single or in combination) or 12.5 µg/mL (BsAbs). Activity of each condition was expressed as % of no antibody control. *p* values of two-tailed Student *t* test to compare the activity of the combinations (black curves) and BsAbs (gray) are indicated in each graph. We conclude that rMabs are as good as the original mMabs in blocking cell–cell fusion, virus entry or virus plaque formation. In addition, the activity of BsAbs was similar using the three in vitro functional assays, suggesting that any of these assays could be used for screening of other future BsAbs candidates.

As we have seen in the fusion assay, the rMAbs, MC2, MC5, DL11 and 1D3 all inhibited virus activity (colored curves in Figure 8A–C). All rMab combinations blocked in a pattern similar to the cell–cell fusion assay (Figure 7): the MC5+DL11 combination was only as good as DL11 alone, while MC2+DL11 and DL11+1D3 were both at least additive (black curves). Furthermore, as in the fusion assay, the MC5/DL11 and MC2/DL11 BsAbs were “indifferent”, while DL11/1D3 BsAb was as good as or better than the combination of DL11+1D3.

(b) Virus plaque assay. One hundred plaque forming units (pfu) of wild-type HSV-2 (333) virus were incubated with two-fold dilutions of rMabs beginning at 6.26 ug/mL (singly or in combination) or 12.5 µg/mL BsAbs. The virus/antibody mix was added to Vero cells for 1 h at 37 °C. Cells were then overlaid with 1% methylcellulose and incubated for 2–3 days until plaques were visible. After fixation and staining with crystal violet, plaques were counted. The activity of the BsAbs was very similar to that observed in the entry assay with MC5/DL11(Figure 8D) and MC2/DL11 (Figure 8E), having an “indifferent” effect, and DL11/1D3 (Figure 8F) as good as the combination. As reference, statistical two-tailed Student *t* test evaluations were performed. The activity of the BsAbs was evaluated against the combination of the two antibodies. *p* values are indicated in each graph.

### 3.8. DL11/1D3 BsAb Has a Synergistic Activity on HSV Virus

**Figure 9:**
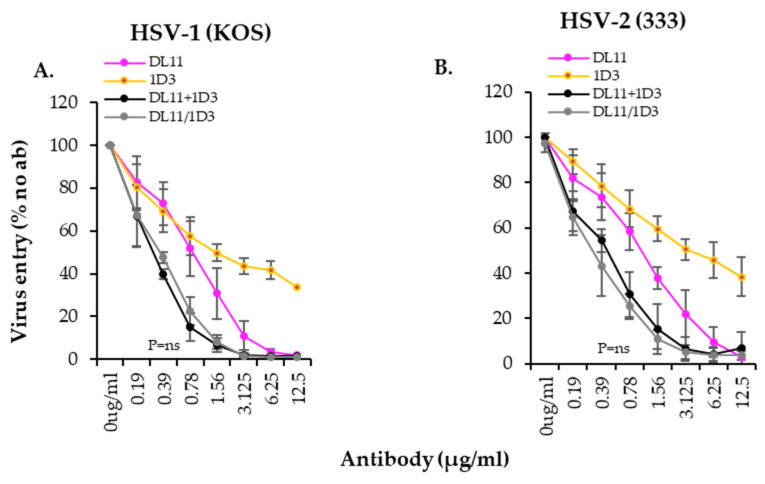
Inhibition of virus entry by recombinant mouse DL11/1D3 BsAb. The inhibitory activity of DL11/1D3 BsAbs (gray lines) was measured against HSV-1 (**A**) or HSV-2 (**B**) using an entry assay. Activity of monospecific antibodies is shown in color. Infectivity of virus under each condition was expressed as % of no antibody control. Standard deviations from at least three independent experiments are shown. *p* values of two-tailed Student *t* test (*p* values) to compare activity of BsAb and DL11+1D3 combinations are indicated in each graph.

During our characterization of the BsAbs, we observed that while the DL11/1D3 BsAb was as good as the combination of the single rMabs (Figure 7F and Figure 8C,F), the activity of the combination was consistently better than a theoretical additive effect against both the HSV-1 and HSV-2 viruses (Figure 9, gray and black curves). This suggested that the two antibodies might have a synergistic activity. This is consistent with reports from Sanna et al. who found that antibody H128 (same competition group as DL11) and antibody H170 (competes with 1D3) were synergistic against the virus [78,79].

**Figure 10:**
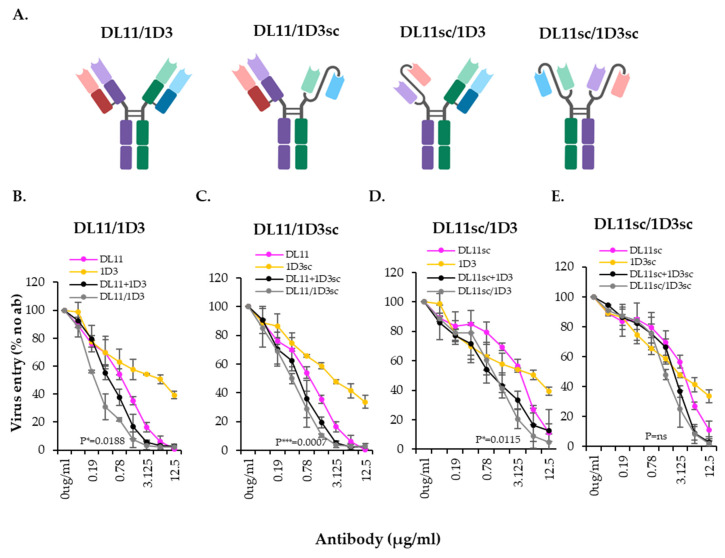
Variations of recombinant mouse DL11/1D3 BsAb. (**A**) Schematic representation of BsAb formats: both antibodies full length (DL11/1D3), one antibody is full-length, and the other is a single chain (DL11/1D3sc and DL11sc/1D3) or both antibodies are single chains linked to the Fc (DL11sc/1D3sc). (**B**–**E**) Inhibitory activity of DL11/1D3 BsAbs against HSV-2 virus in an entry assay. Standard deviations from at least three independent experiments are shown. *p* values of two-tailed Student *t* test (*p* values) to compare DL11 and DL11+1D3 are indicated in each graph. Figure 10B shows their activity against HSV-2 virus using the entry assay. We found that all antibodies had virus-blocking properties, albeit with different potencies, depending on the format. For example, the IgGs for both DL11 and 1D3 were more potent than the single chain versions (compare colored curves in Figure 10B,E). There was a marked difference in the overall activity of BsAbs, especially when BsAbs containing DL11sc were used (Figure 10D,E and Table 3). For example, for DL11/1D3 (Figure 10B), the IC50 was ~0.1 µg/mL, while for DL11sc/1D3 IC50, it was ~1.3 µg/mL. DL11sc/1D3sc had the lowest activity (IC50 ~ 2.6 µg/mL). Despite the 1D3sc being less active than the IgG (compare yellow lines in Figure 10B,C), its activity as part of the BsAb or combination did not change if DL11 was in a full-length format (black and gray curves in Figure 10B,C). The activity of each BsAbs and the respective combination was not statistically significant. However, the combinations were statistically significant compared to DL11 alone (*p* values shown in Figure 10), except for DL11sc+1D3sc, which was the same as DL11sc.

To further understand their mechanism of action and to prepare bispecific antibodies for experiments that will explore the therapeutic potential of these antibodies in a mouse model, DL11 and 1D3 were re-cloned into a mouse IgG2a Fc backbone. We also designed single chain (sc) versions of both DL11 and 1D3 to address the effect that light chain mismatch during BsAb production may have on their activity. Using these DNA constructs, we generated four versions of the DL11/1D3 BsAb (Figure 10A): (a) DL11/1D3, in which both Abs are full length; (b) DL11/1D3sc and DL11sc/1D3, where one Ab is full length IgG and the other is a single chain anchored to an Fc, and (c) DL11sc/1D3sc, where both Abs are single chains. All Abs were purified from supernatants of transfected 293T cells through a Pro G column (for monospecific Mabs) or Nickel column (for BsAbs), using the 6xHis tag located at the C-terminus of DL11 (full length or single chain).

We conclude that rMabs, singly or in combination, have similar in vitro activity as the original Mabs in cell–cell fusion, virus entry and plaque assays, regardless of the nature of Fc (human vs. mouse). To generate a BsAb, it is necessary, but not sufficient, for the initial two antibodies to have an additive inhibitory activity in at least one of these assays. The potency of the BsAb is also dependent on the distance between the two targeted epitopes [76,77] and the ability of the BsAb to engage both paratopes simultaneously.

## 4. Discussion

Entry of HSV into cells occurs in a stepwise cascade that involves the four essential glycoproteins, gD, gH/gL and gB, and a cellular receptor, nectin-1 or HVEM. Multiple Mabs against each of these proteins have been isolated and characterized and the details of their binding location and mechanism of action are known for many [16,26,28,29,30,61,62,65,66,69,71,75,80,81,82,83]. By targeting a specific step in the fusion cascade, a neutralizing antibody effectively stops the infectious cycle. However, the addition of a second antibody that targets a different step in the cascade likely introduces a redundancy that would result in the neutralization of any virus that might escape the action of the first antibody. The two antibodies may either target epitopes located on the same protein or epitopes located on different proteins. The impact of combinations of broadly neutralizing antibodies that target the same protein have been assessed for HIV [84,85,86], Chikungunya virus [87], rabies [88], HCV [89] and herpes viruses [29,79,90,91], with the aim of using combinations of antibodies as immunotherapeutics for the treatment of viral infections.

In the present study, we examined the effects of pairs of Mabs that target gD, with the goal of identifying two with a combined additive effect that could be used as immunotherapeutic BsAbs. By targeting multiple epitopes on gD, BsAbs offer a potential advantage over conventional antibodies by enhancing neutralization and preventing viral entry. By simultaneously targeting two essential epitopes on the protein, a BsAb may overcome viral escape mechanisms and reduce the risk of resistance. Furthermore, a BsAb can inform on the topology of the protein. After binding one paratope to an antigenic epitope, the remaining arm will sample an area of 6–12nm for symmetry-related epitopes [76,92]. If the distance between the target epitopes falls outside of the 6–12nm requirement, the BsAb will only engage one of the arms. This will negatively affect the function of the BsAb relative to that observed using a combination of the two Mabs from which it was designed. These antagonistic BsAbs could be used as tools that inform the distance between epitopes during virus entry. For example, if the BsAb targets epitopes located on two different entry proteins, it may provide invaluable information about the organization of the glycoproteins and/or the fusion unit and the spatial relationship between glycoproteins during virus entry as well as help in vaccine design.

In the gD BsAbs design process, it was essential to identify pairs of antibodies that did not interfere with each other and had an additive inhibitory effect. We had previously shown that a combination of gD and gH/gL Mabs that do not compete for binding to the same epitope but block the same step in the fusion cascade had an antagonistic effect on cell fusion [50]. This suggested that both physical and functional competition would have a negative impact on the activity of the Mabs. Here, we tested the effect of combinations of non-competing Mabs, which were neutralized by blocking different functions in gD [28], to (1) see if there was an enhanced effect on inhibition and (2) study the spatial and mechanistic relationship between gD epitopes for better therapeutic drug design. We found that multiple antibody pairs worked well together, with a blocking activity better than that of the individual antibodies. An improvement in fusion inhibition was confirmed using the Bliss independence model to calculate a theoretical additive curve based on the ability of each antibody to block fusion [50,68]. If the blocking activity of the pair coincided with the theoretical curve, we deemed it additive, and if below the curve, i.e., better inhibition, we deemed it synergistic.

The DL11/1D3 BsAb and the combination of the respective Mabs both showed synergistic activity against the virus. Previously, a combination of Mabs that compete with DL11 and 1D3 (H128 and H170, respectively) had been shown to have a synergistic effect on virus neutralization rates [79]. We hypothesize that other combinations of Mabs from the same competition groups as DL11 (pink community, [28,70]) and 1D3 (yellow community) might have a synergistic effect. Not only do DL11 and 1D3 block different steps in virus entry (see Table 1), but the distance between the two epitopes is optimal for a BsAb to bind both simultaneously. Although decreasing the size of the 1D3 paratope by using a single chain construct reduced its activity, this did not affect its ability to enhance the activity of DL11. This suggested that even with a shorter arm, the DL11/1D3 BsAb could still bind both gD epitopes. However, the activity of DL11 was negatively affected when presented as a single chain, which in turn, affected the efficacy of both the combination of Mabs and BsAb, regardless of the format for 1D3. Notably, there is evidence for the protective effect of DL11 and 1D3 in animal models as the passive transfer of a human gD Mab (HSV8) [93] that was placed in the same community as DL11 by competition assay [78,94] or with murine DL11 or 1D3 Mabs [10] showed 80% protection against death and disease in murine models.

The development of antibody therapeutics to treat infectious disease is hampered by the complexity of the virus, the presence of multiple antigens and strain variation. These therapies may be more viable as combinations of neutralizing antibodies rather than as single antibodies. We propose that the combination of DL11+1D3 or the BsAb DL11/1D3 could be an effective immunotherapeutic for HSV infections. The use of BsAbs singly or in combination with existing medication will enhance efficacy of treatment as well as decrease the development of viral resistance. Data presented here show for the first time the generation, characterization and potential of gD-specific bispecific antibodies in enhancing the inhibition of herpes simplex virus neutralization compared to monoclonal antibodies. Further work will include the optimization of BsAb design, including the design of BsAbs that target epitopes located on two different proteins, as well as the evaluation of the efficacy of BsAbs in animal models of HSV infection.

## Figures and Tables

**Table 1:** Properties of neutralizing gD antibodies used in this study. Sentinel neutralizing antibodies were chosen from each community: MC2 (green), MC5 (blue), DL11 (pink), MC23 (red), 1D3 (yellow). TC = type common; T2 = HSV-2 specific. Abbreviations: Hc, heavy chain; Lc, light chain.

Antibody	Specificity	Community	Epitope/Residues	Isotype (Hc/Lc)	Function
1D3	TC	yellow	10–20 ^a,b,c^	2a/kappa	Blocks HVEM binding
MC2	T2	green	64, 67, 243, 245, 246, 248 ^d,e^	G1/kappa	Stabilizes gD-gH/gL interaction
MC5	TC	blue	54, 75–79 ^d^	G1/kappa	Blocks gH/gL binding
MC23	TC	red	213 ^e^, 216 ^f^	G1/kappa	Blocks gH/gL and nectin binding
DL11	TC	pink	38, 132, 140, 222–224 ^c^	2a/kappa	Blocks nectin binding

a [66]. b [69]. c [61]. d [70]. e [29]. f [71].

**Table 2:** Summary of effects of mouse Mab combinations on blocking cell–cell fusion.

	MC5	MC23	DL11	1D3
**MC2**	antagonism	additive	additive	additive
	**MC5**	additive	antagonism	additive
		**MC23**	antagonism	additive
			**DL11**	additive

**Table 3:** Summary of inhibitory activity (IC50) of recombinant mono- and bispecific antibodies against HSV-1 and HSV-2 in a virus entry assay. Average of three experiments, each performed in duplicate.

	HSV-1	HSV-2
DL11	0.6 ± 0.1	0.5 ± 0.1
1D3	2.3 ± 1.1	1.8 ± 1.1
DL11sc	6.2 ± 0.1	4.1 ± 1.7
1D3sc	9.3 ± 1.5	4.1 ± 1.8
DL11/1D3	0.4 ± 0.1	0.1 ± 0.1
DL11/1D3sc	0.8 ± 0.1	0.2 ± 0.1
DL11sc/1D3	1.5 ± 0.1	1.3 ± 0.4
DL11sc/1D3sc	3.1 ± 0.1	2.6 ± 0.9

## Data Availability

Data are contained within the article.

## Supplementary Materials

The following supporting information can be downloaded at: https://www.mdpi.com/article/10.3390/v17020249/s1, Figure S1: Amino acid sequence of murine gD antibodies.
