# Supplementary material for: Targeting Herpes Simplex Virus Glycoprotein D with Bispecific Antibodies: Expanding Therapeutic Horizons by Searching for Synergy
Source: Viruses. 2025 Feb 12;17(2):249. doi: 10.3390/v17020249 (PMC11860751; doi:10.3390/v17020249)
Supplement: Supplementary file 1 [file viruses-17-00249-s001.zip › viruses-3443519-supplementary.pptx]

## Slide 1
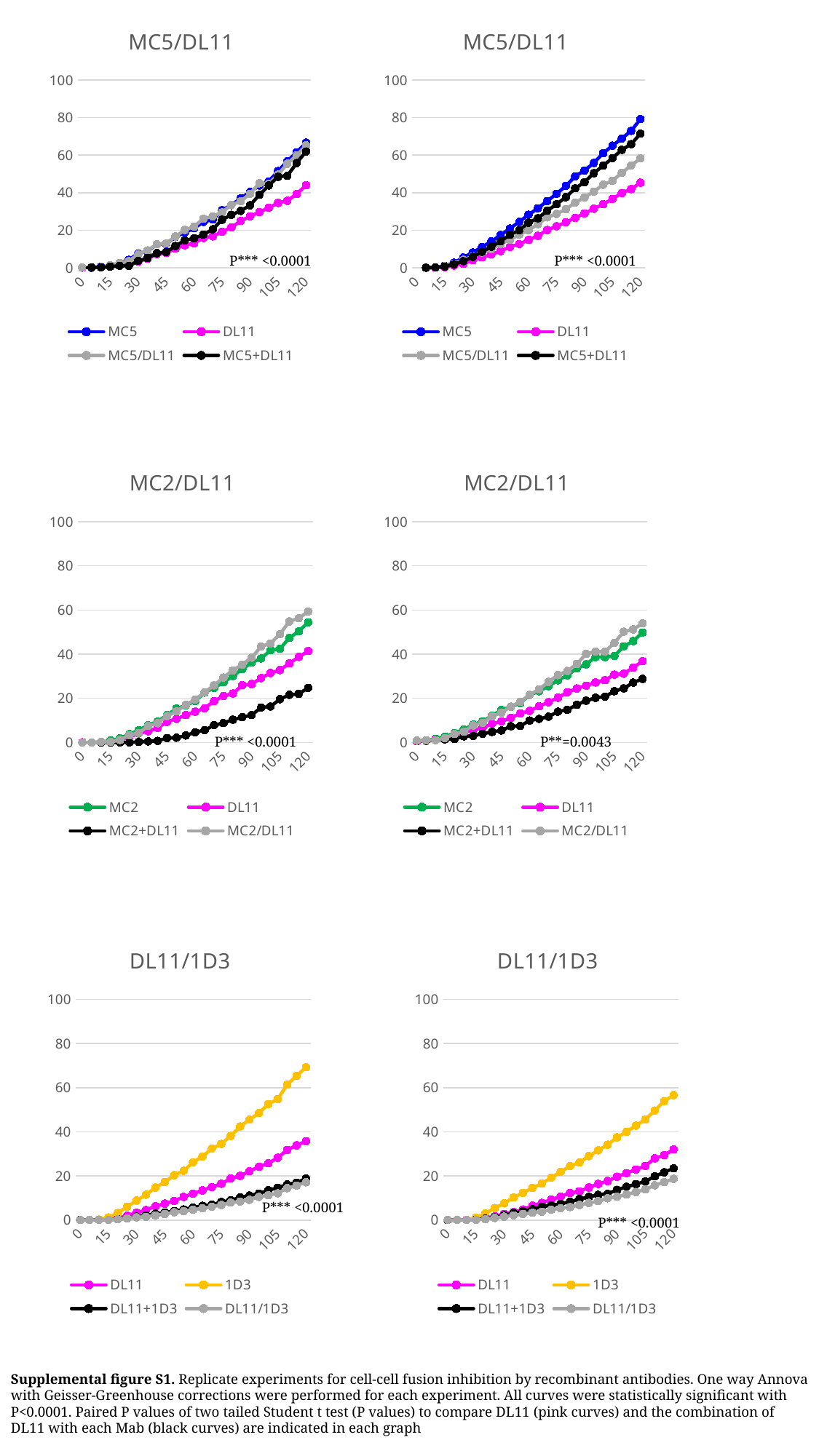

### Chart: MC5/DL11
| Category | MC5 | DL11 | MC5/DL11 | MC5+DL11 |
|---|---|---|---|---|
| 0 | -0.18537380321857813 | 0.028519046649012017 | 0.08148299042574862 | -0.24444897127724588 |
| 5 | 0.0 | -0.1425952332450601 | 0.2037074760643716 | 0.2037074760643716 |
| 10 | 0.5561214096557344 | -0.057038093298024034 | 0.4074149521287432 | 0.24444897127724588 |
| 15 | 0.8474231004277857 | 0.7985333061723365 | 1.3444693420248521 | 0.5703809329802404 |
| 20 | 2.409859441841516 | 1.625585658993685 | 2.607455693623956 | 1.0592788755347322 |
| 25 | 4.21063353025056 | 2.2815237319209616 | 3.5445100835200654 | 0.9777958851089835 |
| 30 | 7.600325931961703 | 3.308209411285394 | 7.1705031574658795 | 3.5852515787329398 |
| 35 | 8.765532695049908 | 4.791199837034019 | 9.207577918109594 | 5.174169892035038 |
| 40 | 12.473008759421472 | 7.186799755551029 | 12.46689753513954 | 7.781625585658994 |
| 45 | 12.65838256264005 | 7.871256875127316 | 12.874312487268282 | 8.352006518639234 |
| 50 | 16.52475045834182 | 10.209818700346302 | 16.663271542065594 | 11.570584640456305 |
| 55 | 18.696272153188023 | 12.00651863923406 | 20.126298635159912 | 14.585455286209005 |
| 60 | 21.053167651252807 | 13.03320431859849 | 21.91892442452638 | 15.644734161743736 |
| 65 | 24.416378081075575 | 15.913628030148706 | 26.115298431452434 | 17.64106742717458 |
| 70 | 25.661030759828886 | 16.712161336321042 | 27.337543287838663 | 20.492972092075778 |
| 75 | 30.58667753106539 | 19.136280301487062 | 29.496842534121004 | 25.54491749847219 |
| 80 | 33.367284579344066 | 21.531880220004073 | 33.489509064982684 | 28.193114687309023 |
| 85 | 36.96883275616215 | 24.9256467712365 | 35.485842330413526 | 30.270930943165613 |
| 90 | 40.33204318598493 | 27.34976573640252 | 39.356284375636584 | 33.28580158891831 |
| 95 | 43.80118150336118 | 29.631289468323484 | 45.01935221022612 | 38.82664493786922 |
| 100 | 45.97270319820737 | 31.884294153595434 | 44.734161743735996 | 43.797107353839884 |
| 105 | 51.480953350987974 | 34.508046445304544 | 49.663882664493784 | 48.44163780810756 |
| 110 | 56.75086575677327 | 35.64880831126502 | 55.408433489509065 | 48.97127724587492 |
| 115 | 61.51762069667957 | 39.29924628233856 | 59.889997962925236 | 55.775106946424934 |
| 120 | 66.60215929924628 | 43.91933183947851 | 65.22713383581178 | 61.927072723568955 |
### Chart: MC5/DL11
| Category | MC5 | DL11 | MC5/DL11 | MC5+DL11 |
|---|---|---|---|---|
| 0 | -0.045071319 | -0.066273932 | -0.050712388 | -0.026414651 |
| 5 | 0.0 | -0.039273441 | 0.0 | 0.005869923 |
| 10 | 0.084840129 | 0.063819342 | 0.106254528 | 0.146748063 |
| 15 | 1.01012779 | 0.326460481 | 0.876599855 | 0.613406903 |
| 20 | 2.669812821 | 1.200294551 | 2.209611205 | 1.851960554 |
| 25 | 5.437722043 | 2.039764359 | 4.24776624 | 3.507278704 |
| 30 | 8.086324832 | 3.966617575 | 6.003380826 | 5.734914299 |
| 35 | 10.99475052 | 5.481099656 | 8.097078 | 8.438013618 |
| 40 | 14.02513389 | 7.088856161 | 9.929968607 | 11.22329185 |
| 45 | 17.376319 | 8.865979381 | 12.68292683 | 14.09955389 |
| 50 | 20.89718437 | 11.09965636 | 14.88529341 | 17.55106833 |
| 55 | 24.39683971 | 12.66077565 | 17.59478387 | 19.91077718 |
| 60 | 28.23585556 | 14.8011782 | 19.89374547 | 24.01972294 |
| 65 | 31.66922955 | 17.01767305 | 23.21419947 | 26.31779761 |
| 70 | 35.51619916 | 20.05891016 | 26.79546003 | 30.38271895 |
| 75 | 39.37642505 | 22.07658321 | 28.63076552 | 33.80488378 |
| 80 | 43.57601145 | 24.2317133 | 31.14223617 | 37.57337403 |
| 85 | 48.72474681 | 26.57830142 | 34.73074137 | 42.29279174 |
| 90 | 51.79755024 | 28.96661757 | 37.45230621 | 45.59462315 |
| 95 | 55.79564134 | 31.42611684 | 40.58681478 | 50.37274008 |
| 100 | 61.00005303 | 33.83406971 | 44.22120261 | 54.39070204 |
| 105 | 65.04586669 | 36.64702995 | 46.36078242 | 58.41746889 |
| 110 | 68.7894374 | 39.79381443 | 50.54817677 | 62.81404085 |
| 115 | 72.9200912 | 41.89494354 | 54.46510505 | 65.81357126 |
| 120 | 79.18765576 | 45.31664212 | 58.21782178 | 71.4017375 |P*** <0.0001
P*** <0.0001
### Chart: MC2/DL11
| Category | MC2 | DL11 | MC2+DL11 | MC2/DL11 |
|---|---|---|---|---|
| 0 | -0.09474182851729038 | 0.07105637138796779 | -0.09474182851729038 | 0.023685457129322594 |
| 5 | -0.11842728564661298 | -0.16579819990525818 | -0.023685457129322594 | 0.04737091425864519 |
| 10 | 0.11842728564661298 | 0.023685457129322594 | 0.001451 | 0.30791094268119373 |
| 15 | 0.9000473709142587 | 0.33159639981051636 | 0.01519 | 0.26054002842254853 |
| 20 | 1.871151113216485 | 1.113216485078162 | 0.04055 | 1.1369019422074846 |
| 25 | 3.74230222643297 | 2.4396020843202275 | 0.04737091425864519 | 3.3159639981051634 |
| 30 | 5.589767882520133 | 4.121269540502132 | 0.23685457129322596 | 4.310753197536712 |
| 35 | 7.839886309805779 | 5.092373282804358 | 0.4737091425864519 | 7.508289909995263 |
| 40 | 9.474182851729038 | 6.631927996210327 | 0.7342491710090004 | 8.739933680720037 |
| 45 | 12.387494078635717 | 9.355755566082426 | 1.989578398863098 | 11.961155850307911 |
| 50 | 15.371861676930365 | 10.705826622453813 | 2.155376598768356 | 14.069161534817622 |
| 55 | 16.745618190431074 | 12.387494078635717 | 3.150165798199905 | 17.05352913311227 |
| 60 | 18.71151113216485 | 13.95073424917101 | 4.642349597347229 | 19.422074846044527 |
| 65 | 22.572240644244435 | 15.513974419706301 | 5.542396968261487 | 22.738038844149692 |
| 70 | 24.680246328754144 | 18.758882046423494 | 7.816200852676457 | 25.864519185220274 |
| 75 | 27.238275698720987 | 21.056371387967786 | 8.76361913784936 | 29.417337754618664 |
| 80 | 30.10421601136902 | 22.16958787304595 | 10.303173851255329 | 32.66224538133586 |
| 85 | 33.18332543818096 | 25.91189009947892 | 11.440075793462814 | 35.29133112269067 |
| 90 | 36.19137849360493 | 26.45665561345334 | 12.387494078635717 | 38.417811463761254 |
| 95 | 38.03884414969209 | 29.156797726196114 | 15.845570819516817 | 43.43912837517764 |
| 100 | 41.75746091899574 | 31.47797252486973 | 16.271909047844623 | 44.88394126006632 |
| 105 | 42.444339175746094 | 32.780672666982476 | 19.587873045949788 | 49.07626717195642 |
| 110 | 47.34722880151587 | 35.83609663666509 | 21.60113690194221 | 54.83183325438181 |
| 115 | 50.35528185693984 | 38.74940786357177 | 22.027475130270012 | 56.30033159639981 |
| 120 | 54.42918048318332 | 41.4021790620559 | 24.72761724301279 | 59.332070108953104 |
### Chart: MC2/DL11
| Category | MC2 | DL11 | MC2+DL11 | MC2/DL11 |
|---|---|---|---|---|
| 0 | 0.6546471170426602 | 0.6180170804126236 | 0.747497627720469 | 0.8122379013743917 |
| 5 | 0.8537655473588621 | 0.7609150366810533 | 0.7792300549960718 | 1.0100809125878762 |
| 10 | 1.6757986674421215 | 1.1774669156284756 | 1.0895140143051005 | 1.063750548430214 |
| 15 | 2.6907771893844314 | 2.0263348536329038 | 1.428979562684298 | 1.9956737784035834 |
| 20 | 4.235819890415991 | 3.1109512585835706 | 1.5571846908894265 | 3.8375830297835867 |
| 25 | 5.900547920046527 | 4.608293285173508 | 2.7836277000622403 | 4.808687134592427 |
| 30 | 8.117685471445917 | 5.606232207903517 | 3.011927719448611 | 7.738834981174815 |
| 35 | 9.575846623200384 | 6.647856785739794 | 3.9951738141153186 | 8.861152774801798 |
| 40 | 12.153111512442988 | 8.480649341373557 | 4.777633230279469 | 11.877314885671431 |
| 45 | 14.70099074555899 | 9.57378554593037 | 5.434627118471129 | 13.507555582764496 |
| 50 | 16.140173661065027 | 11.162906731151836 | 7.359183527707202 | 16.23623822788168 |
| 55 | 17.8485720407726 | 13.153004377238364 | 7.446554837919739 | 18.446998683767486 |
| 60 | 21.518973134572022 | 14.30686583611375 | 9.949865825910415 | 21.544328466334036 |
| 65 | 23.20604650688216 | 16.382855306253646 | 10.670528635709672 | 24.092411766506473 |
| 70 | 25.52883977675064 | 18.253961451733016 | 11.698924566612567 | 27.453804320099586 |
| 75 | 28.1780893201506 | 20.278531125327785 | 13.962773067229906 | 30.503841562337385 |
| 80 | 30.410582917546705 | 22.74913526584838 | 14.79625434917913 | 32.37365698368484 |
| 85 | 33.51087167243156 | 24.45979878988237 | 17.031788545716132 | 35.49695429918271 |
| 90 | 35.36619833277215 | 25.763425061475186 | 18.912470537818734 | 40.11504280306509 |
| 95 | 38.70947993510668 | 27.20777597518545 | 20.254486924403345 | 41.180936055587864 |
| 100 | 38.70947993510668 | 28.20777597518545 | 20.754486924403345 | 41.180936055587864 |
| 105 | 39.164600487720264 | 30.722346363014903 | 23.22675931311029 | 45.09111594069812 |
| 110 | 43.5569398104217 | 31.15672860101831 | 24.482179844296837 | 50.19666962563899 |
| 115 | 45.939575744589675 | 33.92692358709073 | 27.119083330782495 | 51.241901088697745 |
| 120 | 49.81929862152704 | 36.79144857000011 | 28.7890099686757 | 53.93374963012846 |P*** <0.0001
P**=0.0043
### Chart: DL11/1D3
| Category | DL11 | 1D3 | DL11+1D3 | DL11/1D3 |
|---|---|---|---|---|
| 0 | 0.06788866259334692 | 0.06336275175379046 | 0.07241457343290338 | 0.05883684091423399 |
| 5 | 0.07241457343290338 | 0.08599230595157276 | 0.09504412763068568 | 0.07241457343290338 |
| 10 | 0.11314777098891152 | 0.25797691785471827 | 0.13577732518669383 | 0.09957003847024214 |
| 15 | 0.2987101154107264 | 1.2355736591989137 | 0.16745870106358904 | 0.18103643358225843 |
| 20 | 0.9685449196650826 | 3.2179226069246436 | 0.4933242815116542 | 0.39828015388096855 |
| 25 | 2.0276080561212946 | 6.137135098438561 | 0.8825526137135098 | 0.80561212944105 |
| 30 | 3.3627517537904503 | 8.902466621407559 | 1.6157501697216565 | 1.2898845892735913 |
| 35 | 4.589273591310251 | 11.541072640868975 | 2.213170400543109 | 1.552387417967866 |
| 40 | 6.309119710341706 | 14.777098891151844 | 2.9101606698348044 | 2.1724372029871013 |
| 45 | 7.508486082824168 | 17.298031228784794 | 3.5211586331749265 | 2.7834351663272234 |
| 50 | 8.703326544467075 | 20.35754695632496 | 4.145734329033718 | 3.584521384928717 |
| 55 | 10.54989816700611 | 22.344421814890246 | 4.869880063362752 | 4.091423398959041 |
| 60 | 12.016293279022403 | 26.24123104774836 | 5.761484498755374 | 4.720525005657389 |
| 65 | 13.496266123557366 | 28.78479293957909 | 6.512785698121747 | 5.372256166553519 |
| 70 | 15.012446254808781 | 32.46435845213849 | 7.019687712152071 | 6.123557365919892 |
| 75 | 16.48336727766463 | 34.52364788413668 | 8.187372708757637 | 6.902014030323603 |
| 80 | 18.873048200950443 | 38.15342837746096 | 9.0020366598778 | 7.952025345100702 |
| 85 | 20.09051821679113 | 42.43494003168138 | 10.3326544467074 | 8.449875537451913 |
| 90 | 22.131704005431093 | 45.566870332654446 | 11.169947952025344 | 9.223806291016068 |
| 95 | 24.213622991627066 | 48.50418646752659 | 12.08418194161575 | 10.540846345326997 |
| 100 | 25.734329033718037 | 52.60466168816475 | 13.54152523195293 | 11.323828920570264 |
| 105 | 28.28241683638832 | 54.876668929622085 | 14.695632496039828 | 12.183751980085992 |
| 110 | 31.821679112921476 | 61.412084181941616 | 16.13939805385834 | 14.482914686580674 |
| 115 | 33.87644263408011 | 65.3632043448744 | 17.026476578411405 | 15.686806969902692 |
| 120 | 35.79090291921249 | 69.26906539941163 | 18.859470468431773 | 17.153202081918987 |
### Chart: DL11/1D3
| Category | DL11 | 1D3 | DL11+1D3 | DL11/1D3 |
|---|---|---|---|---|
| 0 | 0.0706042090970808 | 0.07694048427245984 | 0.05883684091423399 | 0.1040959493097986 |
| 5 | 0.11767368182846798 | 0.06788866259334692 | 0.06336275175379046 | 0.0814663951120163 |
| 10 | 0.18827789092554878 | 0.17651052274270196 | 0.12219959266802444 | 0.12219959266802444 |
| 15 | 0.38243946594252093 | 1.1269517990495588 | 0.2172437202987101 | 0.16745870106358904 |
| 20 | 0.9649241909934374 | 3.0006788866259333 | 0.6924643584521385 | 0.461642905634759 |
| 25 | 1.78275627970129 | 5.512559402579769 | 1.2808327675944784 | 1.0092781172210907 |
| 30 | 2.8477031002489253 | 7.6080561212944104 | 2.2267481330617787 | 1.5931206155238742 |
| 35 | 3.6831862412310477 | 10.341706268386513 | 3.122878479293958 | 2.2176963113826655 |
| 40 | 4.848155691332881 | 12.342158859470468 | 4.068793844761259 | 2.792486988006336 |
| 45 | 6.589726182394207 | 14.6051142792487 | 4.960398280153881 | 3.3627517537904503 |
| 50 | 7.825299841593121 | 16.58293731613487 | 6.109979633401222 | 3.837972391943879 |
| 55 | 9.337406653088934 | 19.280380176510523 | 6.711925775062231 | 4.734102738176058 |
| 60 | 10.690653994116316 | 21.86467526589726 | 7.46775288526816 | 5.431093007467753 |
| 65 | 12.267481330617787 | 24.48517764200045 | 8.314098212265218 | 5.951572754016746 |
| 70 | 13.073546051142792 | 26.10997963340122 | 9.531568228105906 | 6.915591762842272 |
| 75 | 14.821000226295542 | 29.033718035754696 | 10.531794523647884 | 7.811722109074451 |
| 80 | 16.433129667345554 | 31.663272233537 | 11.500339443312967 | 8.603756505996833 |
| 85 | 17.721656483367276 | 34.19325639284906 | 11.966508259787282 | 9.920796560307762 |
| 90 | 19.680923285811268 | 37.488119484046166 | 13.745191219732972 | 10.604209097080787 |
| 95 | 21.27540167458701 | 40.022629554197785 | 15.112016293279023 | 11.618013125141434 |
| 100 | 22.93460058836841 | 42.828694274722785 | 16.297804933242816 | 12.758542656709663 |
| 105 | 24.570264765784113 | 45.59855170853134 | 17.574111789997737 | 13.908124009957003 |
| 110 | 27.988685222901108 | 49.68997510749038 | 19.837067209775967 | 15.799954740891604 |
| 115 | 29.418420457116994 | 53.85833899072188 | 21.692690653994116 | 17.207513011993665 |
| 120 | 32.03665987780041 | 56.67798144376556 | 23.47589952477936 | 18.73274496492419 |P*** <0.0001
P*** <0.0001
Supplemental figure S1. Replicate experiments for cell-cell fusion inhibition by recombinant antibodies. One way Annova with Geisser-Greenhouse corrections were performed for each experiment. All curves were statistically significant with P<0.0001. Paired P values of two tailed Student t test (P values) to compare DL11 (pink curves) and the combination of DL11 with each Mab (black curves) are indicated in each graph
